# Unraveling rain forest biodiversity: an interview with Thomas Couvreur

**DOI:** 10.1186/s12915-018-0594-4

**Published:** 2018-11-01

**Authors:** Thomas L. P. Couvreur

**Affiliations:** 0000 0001 2097 0141 grid.121334.6 IRD, UMR DIADE, Univ. Montpellier, Montpellier, France

**Keywords:** Biodiversity, Tropical rain forest, Taxonomy, Ecosystems, Palms

## Abstract

Thomas Couvreur is a researcher and botanist at the Institut de Recherche pour le Développement (IRD), based in Montpellier, France, studying tropical biosystems. He is using diverse approaches—from taxonomy, molecular phylogenetics, phylogeography, to modeling species distribution—to understand the evolution and resilience of biodiversity in rain forests. In this interview, Thomas describes the ongoing research in his lab, the most urgent challenges and opportunities in biodiversity research, and the importance of knowing how to code.

## What are the questions driving your research?

My main interest lies in understanding the diversity, evolution, and resilience of tropical rain forest biodiversity, one of the most complex and diverse ecosystems on the planet. Rain forests are important global climate regulators and provide subsidence such as food and shelter to millions of people across the tropics.

The first question that drives my research is what is tropical biodiversity made of? How many species are there and how can we identify them? How are species distributed? Are they common, rare, or scattered across the vegetation? Looking for answers to these questions is the task of taxonomy and provides the fundamental framework for my research, and research in biodiversity in general. I mainly focus on two wonderful tropical rain forest plant families, palms [1] and the Soursop family Annonaceae [2].

A second series of questions focuses on understanding the evolution of this biodiversity over long (millions of years) and shorter (thousands of years) time scales. How has tropical biodiversity evolved through time? What are the important biotic or abiotic components that explain the high level of species diversity under the tropics, and in rain forests in particular?

Finally, I use my research data to address pressing questions in conservation, such as how threatened is tropical biodiversity? How will species respond to ongoing climate changes?

My research has an impact in tropical biodiversity conservation and provides fundamental data towards assessing the influence of ongoing climate change on tropical rain forest biodiversity.

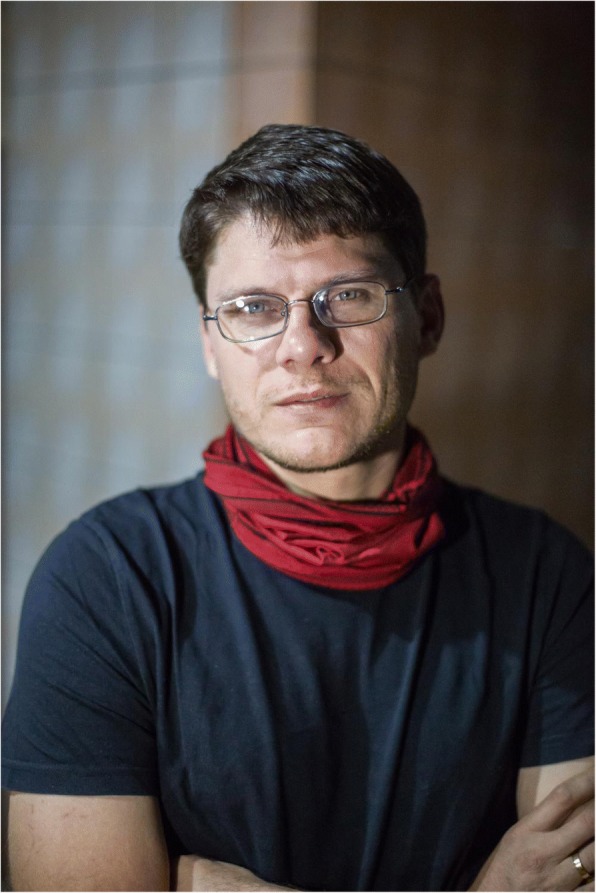



## Given the increasing pressures on biodiversity and ecosystems, how has the field developed over the years? What are the most urgent challenges?

Without a doubt the large scale digitization of biodiversity data deposited in herbaria and museums worldwide. I think the recent fire that devastated the natural history collection of Brazil’s National Museum is a stark (and shocking) reminder of how fragile our biodiversity collections are; 200 years of science can go up in flames, literally, in a few hours. Although access to real specimens is irreplaceable, digitization provides at least some record of the specimens and the data they contain. For example, over the past decade, numerous international herbaria have scanned and digitized millions of plant specimens, making them available online (e.g. [3–5]). Not only is the image of the specimen visible but the associated data of each specimen (location, collection date, habitat, etc.) is also painstakingly captured and entered into databases. These efforts are even more important in regions of high biodiversity concentration such as the tropics, and rain forests in particular, where our understanding of biodiversity remains limited. Open access online databases can and will accelerate our basic understanding of species taxonomy and distribution patterns, a prerequisite for any biodiversity study. This type of large scale endeavor, integrated with data from other fields such as earth and social sciences, opens the way to large scale biodiversity syntheses providing fundamental data towards understanding the current state, past evolution, and future of biodiversity.

## Looking back, is there a project that your lab pursued that stands out for you as particularly inspiring, tough, or simply memorable?

In 2013, we synthesized for the first time our knowledge about tropical African plant biodiversity. Leaning on the massive digitization effort of plant specimens for numerous participating herbaria and a consortium of several African botany experts, we generated a high quality open access mega database called RAINBIO. RAINBIO contains to date over half a million African plant specimens representing distribution information for over 25,000 plant species [6]. This biodiversity synthesis exercise allowed us to address numerous questions about the current state and conservation of African tropical plant biodiversity [7–9], and especially rain forests. We are now using this database and modeling approaches to understand how tropical vegetation will respond to climate change across tropical Africa by the end of this century. The project was tough because we were dealing initially with thousands of records that needed to be sorted, quality checked, and validated. We generated automated scripts to deal with this mass of information, which was then checked by the experts to ensure the database is as accurate as possible. This was inspiring because it highlighted the vital role of taxonomists and experts. The availability of masses of online biodiversity data gives the impression that all is known and perfect, but the reality is that biodiversity data need to be constantly updated, curated, and validated, which is part of the taxonomists’ work. Now more than ever we need to continue efforts to discover and describe biodiversity around us, especially in high biodiversity regions such as the tropics.

## Is there a paper or a scientist that inspired you, or was seminal for your research?

*Origin and evolution of tropical rain forests* published in 2000 by Robert J. Morley [10] is for me the seminal publication for my research. Based on an extensive review of fossil data, this book provides a wealth of information about rain forests and their evolution. It puts forth a series of hypotheses about rain forest evolution that we are now able to test using different complementary approaches such as DNA sequencing and dated molecular phylogenies.

## If you could, what would you tell your younger self?

I guess I would tell my younger self to learn how to code properly! As I said, synthesizing the massive amount of biodiversity data depends on how well we can analyze these data, which depends on one’s ability to code and script. Nowadays, I depend on my PhD students and post docs to do this (for which I greatly admire them!), but I wish I also had that ability.

**Website:** http://www.couvreurlab.org/

**Twitter:** @tlpcouvreur
